# The Role of Doxycycline and IL-17 in Regenerative Potential of Periodontal Ligament Stem Cells: Implications in Periodontitis

**DOI:** 10.3390/biom13101437

**Published:** 2023-09-24

**Authors:** Ivana Okić Đorđević, Tamara Kukolj, Milena Živanović, Sanja Momčilović, Hristina Obradović, Anđelija Petrović, Slavko Mojsilović, Drenka Trivanović, Aleksandra Jauković

**Affiliations:** 1 Group for Hematology and Stem Cells, Institute for Medical Research, University of Belgrade, 11000 Belgrade, Serbia; 2 Group for Neuroendocrinology, Institute for Medical Research, University of Belgrade, 11000 Belgrade, Serbia

**Keywords:** doxycycline, periodontal ligament MSCs, Interleukin 17, regenerative properties

## Abstract

Periodontitis (PD) is a degenerative, bacteria-induced chronic disease of periodontium causing bone resorption and teeth loss. It includes a strong reaction of immune cells through the secretion of proinflammatory factors such as Interleukin-17 (IL-17). PD treatment may consider systemic oral antibiotics application, including doxycycline (Dox), exhibiting antibacterial and anti-inflammatory properties along with supportive activity in wound healing, thus affecting alveolar bone metabolism. In the present study, we aimed to determine whether Dox can affect the regenerative potential of periodontal ligament mesenchymal stem cells (PDLSCs) modulated by IL-17 in terms of cell migration, osteogenic potential, bioenergetics and expression of extracellular matrix metalloproteinase 2 (MMP-2). Our findings indicate that Dox reduces the stimulatory effect of IL-17 on migration and MMP-2 expression in PDLSCs. Furthermore, Dox stimulates osteogenic differentiation of PDLSCs, annulling the inhibitory effect of IL-17 on PDLSCs osteogenesis. In addition, analyses of mitochondrial respiration reveal that Dox decreases oxygen consumption rate in PDLSCs exposed to IL-17, suggesting that changes in metabolic performance can be involved in Dox-mediated effects on PDLSCs. The pro-regenerative properties of Dox in inflammatory microenvironment candidates Dox in terms of regenerative therapy of PD-affected periodontium are observed.

## 1. Introduction

Periodontitis (PD) is a common, chronic inflammatory disease caused by bacterial infection of tooth-supportive tissue that affects 5–20% of middle-aged (35–44 years) adults in Europe, and up to 40% of older people (65–74 years) [1]. Although it is a largely preventable condition, if left untreated, PD leads to teeth loss due to the destruction of periodontal tissue through extracellular matrix (ECM) degradation and alveolar bone resorption [1]. PD treatment usually considers local causal therapy of the affected periodontium, such as cause-related periodontal therapy, including subgingival instrumentation with or without adjunctive use of chemical agents, local or systemic antibiotics, and lasers or performing surgical methods. Generally, the success of PD therapy depends on the level of proinflammatory mediators, enzymes and cytokines in all development stages of the disease [2]. However, due to its infectious nature, PD treatment in some cases includes systemic treatment with antibiotics [3]. Regarding oral antimicrobial agents, PD has primarily been treated with tetracycline drug family antibiotics, including doxycycline (Dox) [4]. Aside from its antibacterial and anti-inflammatory properties, Dox also exhibits its protective activity in wound healing by inhibiting the activity of matrix metalloproteinases (MMPs) [5], a family of ECM-degrading enzymes that have a key role in the physiological remodeling of the periodontal tissue [6]. While elevated levels of MMPs facilitate cell migration, their tissue inhibitors TIMPs act as local control of the MMP activity in tissues both as inhibitors of the active MMPs and proMMPs [6]. Moreover, the imbalance between MMPs and their inhibitors, TIMPs, has been associated with PD [6]. One of the principal MMPs involved in the digestion of native fibrillar collagen implicated in periodontal tissue degradation is MMP-2, produced by various types of stromal and immune cells [6].

Successful regeneration of damaged periodontal tissue depends on the appropriate functionality of periodontal ligament mesenchymal stem cells (PDLSCs), a small population of mesenchymal stromal/stem cells (MSCs) within the periodontal ligament that also express MMPs. Due to their ability to repair damaged periodontal ligament through prominent proliferative and migratory capacity, as well as differentiation to fibroblasts, osteoblasts, or cementoblasts in response to specific microenvironment signals, including signals from the extracellular matrix of other dental-derived MSCs [7], PDLSCs have strong potential to be used in tissue engineering and reconstructive dentistry [8,9].

Based on previous research, proinflammatory cytokines are considered important mediators of PDLSCs’ regenerative capacity [10]. Namely, the inflammatory microenvironment developed in response to bacteria-derived factors leads to the homing and recruitment of nonresident leukocytes to periodontal space, initiating adaptive immunity response through the secretion of proinflammatory cytokines and chemokines. One of the proinflammatory cytokines implicated in various human inflammatory diseases [11,12] produced by Th17 cells [10], present in chronic periodontal lesions [10,13] and playing a central role in PD, is Interleukin 17 (IL-17). Through induction of secondary inflammatory mediators and influencing widespread cellular functional properties such as cell migration and differentiation [14], IL-17 is involved in immune defense mechanisms that have been highly investigated in recent years [15]. Moreover, IL-17 has also been implicated in the regulation of osteogenic potential and MMP expression in MSCs, including PDLSCs [16]. Namely, our previous studies showed negative effects of IL-17 on the osteogenic potential of PDLSCs, evidenced by decreased ALP expression and extracellular Ca deposition in PDLSCs [17], along with its stimulatory effects on both gene and protein expression of MMP-2 in these cells [16]. Nevertheless, to our knowledge, the effects of Dox on IL-17-modulated properties of PDLSCs in terms of cell migration, osteogenic potential and expression of MMP-2 have not been studied yet. Through this system, we established an in vitro model of PD where we induced IL-17 as a cytokine to mimic the inflammatory environment. The aim of this study was to determine if Dox influences the mobility, regenerative properties and metabolic capacity of PDLSCs in an inflammatory microenvironment.

Our findings indicate Dox as a potent modulator of energy-demanding stem cell activities, such as differentiation and migration, and energy-stimulative stem cell activities, such as mitochondrial-based metabolism in PDLSCs treated with IL-17. These data indicate pro-regenerative properties of Dox in the inflammatory microenvironment of PD-affected periodontium, making it a good candidate to develop alternative regenerative strategies for the improvement of periodontal tissue function.

## 2. Materials and Methods

### 2.1. Isolation and Characterization of PDLSCs

Periodontal tissue samples were obtained from 4 healthy individuals distributed equally by gender (18–25 years old) through the extraction of 3rd molars at the Department of Oral Surgery of the Faculty of Dental Medicine, University of Belgrade, in accordance with the guidelines set by the local ethical committee. After the extraction, PDL tissue was carefully separated from the tooth root surface as previously described [18] and minced into small pieces. Tissue samples were then transferred to a 25 cm^2^ flask and cultured in growth medium (GM)—Dulbecco’s modified Eagle’s medium (DMEM; Capricorn Scientific, Ebsdorfergrund, Germany) supplemented with 10% fetal bovine serum (FBS; Capricorn Scientific) and 1% penicillin/streptomycin (Gibco, Life Technologies, Carlsbad, CA, USA). Upon reaching confluence, cells were detached using 0.25% Tripsyn/EDTA (Capricorn Scientific, Ebsdorfergrund, Germany) and further expanded in growth medium (GM). Throughout this study, cells were grown in a humidified atmosphere at 37 °C and 5% of CO_2_. Passages 3–6 were used for all conducted experiments.

To verify the MSCs’ nature of isolated cells, the minimal criteria for defining MSCs by the International Society for Cellular Therapy were followed [19]. Briefly, the expression of positive and negative mesenchymal stromal markers, as well as multilineage differentiation potential, was determined, as reported before [19]. Namely, tri-lineage differentiation potential was confirmed through the cultivation of cells in specific differentiation media (see Section 2.7). The expression of the set of markers characteristic for MSCs was demonstrated using flow cytometry analysis.

### 2.2. Flow Cytometry Analyses

PDLSCs were grown in GM and detached using 1 mM EDTA. Upon harvesting, cells were washed in cold Dulbecco’s phosphate-buffered saline (PBS; Capricorn) supplemented with 0.5% bovine serum albumin (BSA; Sigma-Aldrich, Saint Louis, MO, USA) and separated into aliquots of 2 × 10^5^ cells. Cells were then labeled for 30 min at +4 °C in the dark with monoclonal antibodies specific for human antigens including CD45, CD235a, CD90, CD44H, CD73 (all from R&D Systems, Minneapolis, MN, USA), CD-105, CD29 (Invitrogen, Waltham, MA, USA), CD34 (Dako Cytomation, Glostrup, Denmark) and CD11b (Biosource, Camarillo, CA, USA), each of which was conjugated with either FITC or PE. To determine the level of nonspecific binding, FITC- and PE-conjugated isotype control antibodies (R&D Systems) were used. The analysis was conducted using a CyFlow SL flow cytometer (Partec, Münster, Germany).

### 2.3. Viability Assay (MTT)

To determine the effects of IL-17 and Dox on the proliferation capacity of PDLSCs, MTT (3-(4,5-dimethylthiazol-2-yl) 2,5-diphenyltetrazolium bromide) assay was used, based on the ability of metabolically active cells to convert soluble MTT into an insoluble formazan. Cells were seeded in 96-well plates (2000 cells/well), cultivated in standard conditions in GM for 24 h, and then treated with recombinant human (rh) IL-17 (R&D Systems, Abingdon, UK) (50 and 100 ng/mL) and/or Dox (100 ng/mL) for another 24 h, 48 h and 72 h. After each indicated period, MTT solution (Sigma-Aldrich, St. Louis, MO, USA) (5 mg/mL) was added to the cell culture and incubated for an additional 2 h. Optical density at 540 nm of formazan crystals dissolved in isopropanol was detected using an automatic reader for microtiter plates (RT-6100, Rayto Life and Analytical Sciences, Shenzhen, China).

### 2.4. Migration

To analyze the influence of IL-17 and Dox treatment on PDLSC migration, a scratch assay was conducted. When PDLSCs grown in GM in 24-well plates reached confluence, a scratch was made in the cell monolayer over the diameter of the wells using a sterile pipette tip. Cells were then treated with corresponding factors and incubated for an additional 24 h. Afterward, cells were fixed in methanol, stained with 0.3% crystal violet and analyzed for cell migration into the scratched area using light microscopy (Olympus, Tokyo, Japan). NIH-ImageJ Software V 1.8.0. was used for quantification.

### 2.5. Immunofluorescence

For the purpose of immunofluorescent staining, cells were seeded on the top of rounded coverslips in 24 well plates at 3 × 10^3^ cells/well. After 24 h, PDLSCs were treated and incubated for another 24 h with IL-17 (50, 100 ng/mL) and/or Dox (100 ng/mL). Following these treatments, coverslips were fixed with 4% formaldehyde in PBS. Upon permeabilization with 0.1% Triton X-100 in PBS and blocking with 1% BSA/PBS, cells were labeled overnight with the following primary antibodies: mouse anti-MMP-2, mouse anti-vimentin (both Santa Cruz Biotechnology, Dallas, TX, USA), rabbit anti-Ki67, rabbit anti-IL-6 (both Abcam, Cambridge, UK) and phalloidin. Afterward, cells were washed with PBS and stained with corresponding FITC-conjugated secondary antibodies (Cell Signaling Technology, Danvers, AS, USA) and 0.5 ng/mL of DAPI nuclear dye (Sigma-Aldrich). Mounted samples were examined using an epifluorescence microscope (Olympus, Tokyo, Japan).

### 2.6. Zymography

To analyze the activity of MMP-2, cells were seeded in 24-well plates and grown in GM. Upon reaching confluence, cells were treated with IL-17 and/or Dox and cultivated for an additional 24 h in a serum-free medium. Afterward, the conditioned medium was collected and subjected to sodium dodecyl sulfate–polyacrylamide gel electrophoresis (SDS-PAGE) using a mini protein system (Bio-Rad, Richmond, CA, USA), as previously shown [20]. SDS-PAGE was performed in 8% polyacrylamide gels containing 0.1% gelatin under non-reducing conditions. The gel was then washed in 2% Triton X-100 for 30 min and incubated in 100 mM Tris-HCl, pH 8.5, with 10 mM CaCl2. After 24 h, gels were stained with Coomassie Brilliant blue R-250 for 30 min, causing the appearance of transparent bands inside the colored gel, which corresponds to the activity of MMP-2. Following the staining, the gel was scanned using the ChemiDoc Imaging System (Bio-Rad), and the activity of MMP-2 was quantified using NIH-Image J software V 1.8.0.

### 2.7. Differentiation

To evaluate the effects of IL-17 and Dox on in vitro differentiation of PDLSCs, cells were cultured until sub-confluence in GM. Henceforth, cells were grown in specific differentiation media, while a portion of cells was cultured in GM as a control.

The influence of the above-stated factors on PDLSCs’ potential for osteogenic differentiation was examined through cultivation of cells in 24-well plates in GM supplemented with 10 nM dexamethasone (Sigma-Aldrich, St. Louis, MO, USA), 50 μM ascorbic acid-2-phosphate (Sigma-Aldrich) and 10 mM β-glycerophosphate (AppliChem, Darmstadt, Germany) for 7 and 21 days. At the same time, cells were treated with rising concentrations of IL-17, Dox and a combination of both factors. After 7 days, early osteogenesis was detected through the activity of alkaline phosphatase (ALP) by staining fixed cells with 5-bromo-4-chloro-3-indolyl phosphate/nitro blue tetrazolium (Sigma-Aldrich). Mineralization of extracellular matrix was visualized after 21 days using Alizarin red stain (Merck, Darmstadt, Germany). For both types of staining, cells were examined using a light microscope (Olympus, Tokyo, Japan), while quantification of results by densitometry analysis was performed using NIH-Image J software V 1.8.0.

The adipogenic and chondrogenic potential of PDLSCs was assessed by the cultivation of cells for 21 days in 96-well plates in adipogenic medium (GM supplemented with 1 µM dexamethasone, 10 µg/mL insulin and 100 µg/mL isobutyl-methylxanthine (IBMX) (all Sigma-Aldrich)) and chondrogenic medium (GM supplemented with 2 ng/mL transforming growth factor-β1 (TGF-β1; R&D Systems), 10 nM dexamethasone and 50 μM ascorbic acid-2-phosphate (both Sigma-Aldrich)), respectively. Adipogenic differentiation was visualized by staining lipid droplets with Oil Red O (Merck Chemicals, Burlington, MA, USA). The potential of PDLSCs for chondrogenesis was evaluated using Safranin O stain (Merck Chemicals).

### 2.8. Quantitative Real-Time PCR

Total RNA was extracted using TRIzol reagent (AppliChem GmbH, Darmstadt, Germany) from harvested cells grown in 6-well plates until confluence and treated for 24 h with corresponding treatments. The concentration of isolated RNA was measured with a spectrophotometer (Nanodrop, Thermo Fisher Scientific, Waltham, MA, USA). cDNA was synthetized using the High-Capacity cDNA Reverse Transcription Kit (Thermo Fisher Scientific) with 1 μg of total RNA of each sample. Expression of target genes was analyzed using generated cDNA, appropriate primers and Fast Green Kit (Applied Biosystems, Foster City, CA, USA) in triplicate in Mic qPCR cycler (Bio Molecular Systems, Upper Coomera, Australia). GAPDH was used as an internal control. The comparative ΔΔCt method was used to quantify the relative gene expression [21]. The primer and probe sequences are listed below (Table 1).

### 2.9. Analyses of Cellular Bioenergetics—Mito Stress Assay

Expanded PDLSCs were trypsinized, re-suspended in medium and plated at the density of 4 × 10^3^ cells per well in 96-well XF plates (Agilent, Santa Clara, CA, USA) in GM. One day after, adherent cells were treated with IL-17 with or without Dox for the next 72 h. When treatment was stopped, cell efflux was analyzed by Seahorse according to the following protocol: On the day of measurements, the media were replaced with pre-warmed XF Base (with adjusted pH 7.4) DMEM (Agilent, Santa Clara, CA, USA). Before the evaluation of oxygen consumption rate (OCR) values, the cells were incubated for 1 h at 37 °C in the absence of CO_2_ and 20% of O_2_. OCR and extracellular acidification rate (ECAR) were measured using the Mito Stress assay, where OCR, as an indicator of mitochondrial respiration, and ECAR, as an indicator mainly of lactate and bicarbonate production, were determined simultaneously. During the assay, the following modulators of mitochondrial electron transport chain complexes were applied: 1.5 µM oligomycin (inhibitor of ATP synthase), 0.5 µM rotenone (inhibitor of NADH:ubiquinone oxidoreductase), antimycin A (inhibitor of coenzyme Q: cytochrome c—oxidoreductase) and 1 µM FCCP as a general uncoupling agent that disrupts the mitochondrial membrane potential. After analysis, the XF medium was discarded, and cell viability was checked by light microscopy. Cells were trypsinized, pooled (from all wells/group) and counted, and all values obtained by Mito Stress assay were normalized to the average number of cells/groups. Experiments were performed in 3 technical replicates in Agilent Seahorse XF HS Mini Analyzer. Parameters were determined according to the instructions: https://www.agilent.com/cs/library/usermanuals/public/XF_Cell_Mito_Stress_Test_Kit_User_Guide.pdf (accessed on 15 March 2023).

### 2.10. Statistical Analyses

Results from three independent experiments are shown. Data are given as means ± SEM. Statistical significance was evaluated by combining the non-parametric and ANOVA (Kruskal–Wallis test) combining Excell and GraphPad Prism. Differences were considered to be significant at a value of * *p* < 0.05, ** *p* < 0.01, *** *p* < 0.001.

## 3. Results

### 3.1. Phenotypic Properties and Multilineage Differentiation of PDLSCs

**Figure 1:**
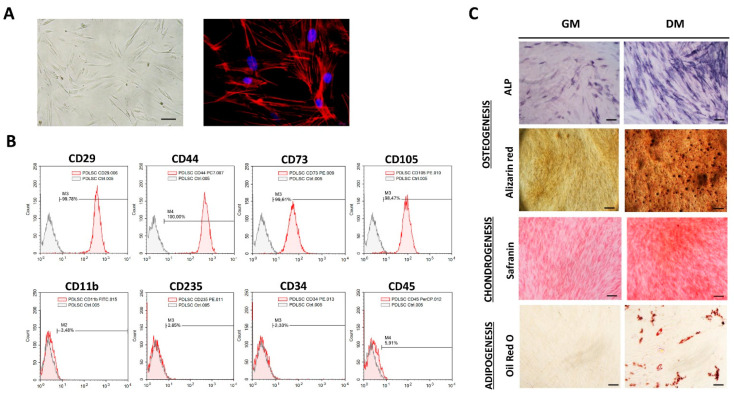
Morphology, immunophenotype and differentiation potential of PDLSCs: (**A**) Adherent PDLSCs with fibroblast-like shape grown in GM under standard conditions for 3 days (scale bars: 50 µM); Florescent images of TRITC-conjugated phalloidin labeled F-actin (red) merged with DAPI (4′,6-diamidino-2-phenylindole) nuclear staining (blue) (scale bars: 10 µM). (**B**) Immunophenotypic characteristics of PDLSCs estimated by flow cytometry. Representative histograms present percentages of cells positive (empty peaks) for mesenchymal markers (CD29, CD44, CD73 and CD105) and hematopoietic markers (CD11b, CD235, CD34 and CD45) in comparison to isotype control (shaded peaks). (**C**) Tri-lineage differentiation potential of PDLSCs. Cells were cultured in growth (GM) and differentiation medium (DM). Osteogenic differentiation was determined by positive staining for ALP activity and extracellular matrix mineralization with Alizarin red; scale bars: 50 m; positive staining of proteoglycans by Safranin O confirmed chondrogenic differentiation of PDLSCs; scale bars: 50 m; Oil Red O staining of intracytoplasmic lipid droplets demonstrated adipogenic differentiation; scale bars: 20 µm.

MSCs identity of isolated cells was determined following the minimal criteria for MSCs identification set by the International Society for Cellular Therapies [19]. Isolated adherent PDLSCs of each donor kept fibroblast-like morphology during long-term cultivation with a similar cytoskeleton organization of F-actin (Figure 1A). Besides the morphology, flow-cytometry analyses revealed that PDLSCs exhibited typical immunophenotype of MSCs (Figure 1B) with high expression of MSCs surface markers (CD29, CD44, CD73 and CD105), along with the low rate of hematopoietic (CD11b, CD235, CD34 and CD45) cell markers expression. Namely, more than 99% of PDLSCs expressed CD29, CD44, CD73 and CD105, while less than 6% of PDLSCs expressed CD11b, CD235, CD34 and CD45. Regarding MSCs’ differentiation potential, we demonstrated that isolated cells of each donor have tri-lineage differentiation capacity (Figure 1C), while no spontaneous differentiation was observed. Specifically, PDLSCs cultivated in osteogenic medium for 7 days showed higher expression of ALP compared to cells cultivated in GM. Also, matrix mineralization was evidenced for PDLSCs cultured in OM for three weeks as determined by Alizarin red staining of extracellular Ca depositions. Similarly, PDLSCs showed the potential to differentiate into the cells of chondrogenic lineage when cultured in a chondrogenic medium for three weeks, as determined by higher Safranin O staining of proteoglycans. At the same time, intracellular lipid droplet formation was shown by Oil red staining for PDLSCs of each donor upon 3 weeks of cultivation in an adipogenic medium.

### 3.2. Dox Inhibits Cell Migration of PDLSCs Treated with IL-17

**Figure 2:**
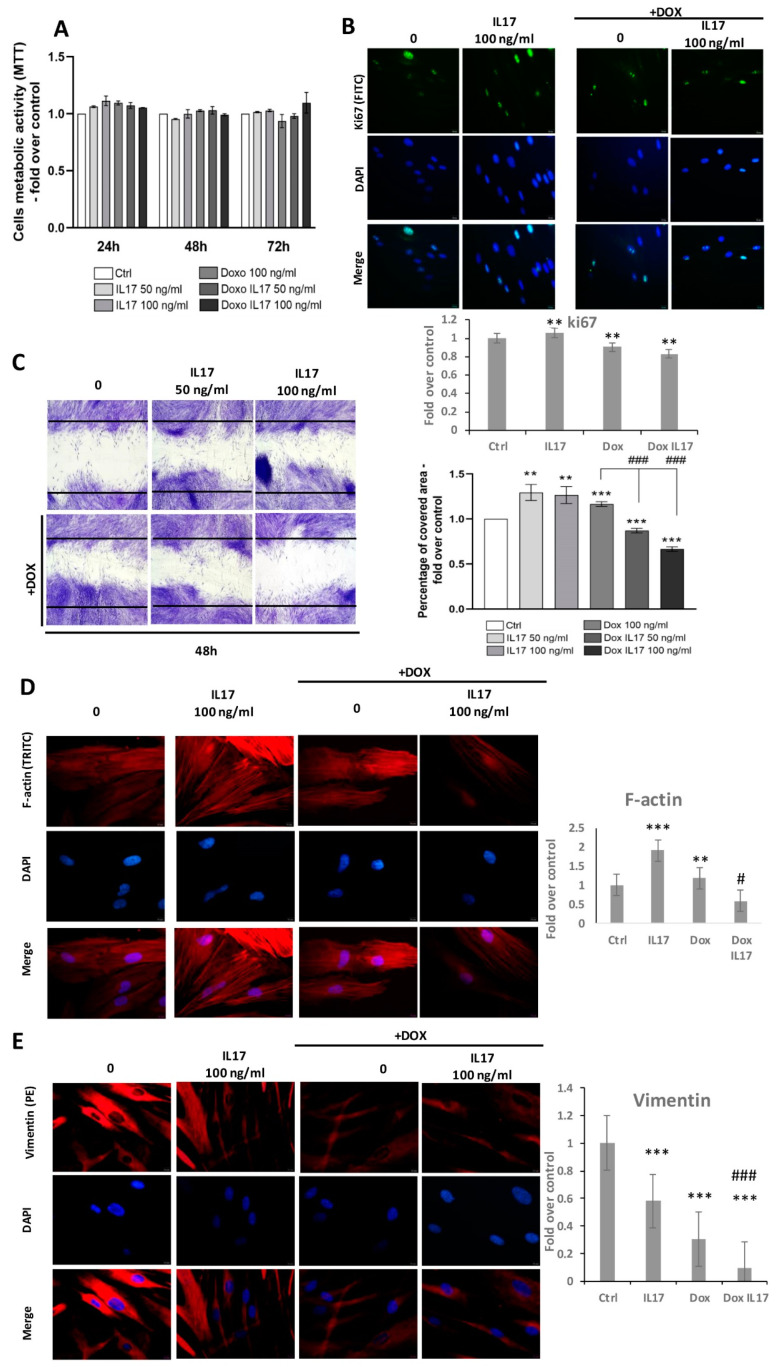
Effects of IL-17 and Dox on PDLSCs growth and migration: (**A**) Proliferation of PDLSCs determined by MTT test after 24, 48 and 72 h of incubation with IL-17 (50 and 100 ng/mL) and Dox (100 ng/mL). Absorbance values were normalized to the control level and presented as the mean ± SEM from three independent experiments. (**B**) Expression of proliferation marker Ki67 in PDLSCs. Cells were labeled with Ki-67 primary antibody and corresponding secondary FITC-conjugated antibody along with DNA stain DAPI. Representative images of immunofluorescence microscopy obtained in three independent experiments are shown. Scale bars: 20 μm. Graphical presentation of ki67 protein expression as analyzed by Image J, relative protein expression normalized to control. Results in graphs are presented as mean ± SEM from at least three independent experiments (n = 3). Statistically significant differences: ** *p* < 0.01 compared to control. (**C**) Migration of PDLSCs analyzed by scratch assay: after a scratch was made in the confluent monolayer, cells were incubated in GM with 0, 50 and 100 ng/mL IL-17 and Dox 100 ng/mL for 24 h. Representative pictures from four experiments (each performed in triplicate) are presented. The graph represents the percentage of the scratch area covered with migrating cells compared to corresponding nontreated control (100%). Results in graphs are presented as mean ± SEM from at least three independent experiments. Significant difference versus corresponding control indicated by darts and calculated by *t*-test: ** *p* < 0.01, *** *p* < 0.001, ### *p* < 0.001. (**D**) PDLSCs were immunostained with phalloidin and (**E**) vimentin primary antibody, corresponding TRITC-conjugated secondary antibody and DAPI. Representative images of three independent experiments are shown. Scale bars: 10 μm. Graphical presentation of phalloidin and vimentin protein expression as analyzed by Image J, relative protein expression normalized to control. Results in graphs are presented as mean ± SEM from at least three independent experiments. Statistically significant differences: ** *p* < 0.001, *** *p* < 0.001 compared to control, # *p* < 0.05 ### *p* < 0.001 compared to Dox.

A comparison of PDLSCs viability performed by MTT test revealed equivalent metabolic activity between treatments with IL-17 and Dox following 24 and 48 h (Figure 2A). With a minimal increase in groups treated with IL-17 and Dox, this trend was also maintained after 72 h, with no statistical significance (Figure 2A). Further, proliferative abilities were also tested by the expression of intracellular proliferation marker Ki67 (Figure 2B). Immunostained PDLSCs constitutively express Ki67, and all tested groups predominantly express Ki67 in the nucleus of PDLSCs. Interestingly, a slight decrease in Ki67 expression was determined in cells treated with Dox and Dox+IL-17, while treatment with IL-17 only demonstrated a similar expression of Ki67 as in the nontreated control group of PDLSCs.

To determine whether IL-17 and Dox affect the motility of PDLSCs, a scratch assay was performed. Namely, after reaching confluence, PDLSCs were allowed for 24 h to migrate into the scratch of wells in the presence of IL-17 (0, 50,100 ng/mL) with or without Dox (100 ng/mL). As shown in Figure 2C, a significant increase in the percentage of migrating PDLSCs was noticed when cells were treated with IL-17 50 and 100 ng/mL. As expected, treatment of PDLSCs with Dox also increased the migration ability of PDLSCs. Interestingly, IL-17 significantly and dose-dependently inhibited the motility of PDLSCs treated with Dox. This result reveals that the wound-healing ability of Dox can be altered in inflammatory conditions by IL-17.

To further study the migratory capabilities of PDLSCs under the influence of IL-17 and Dox, the cytoskeleton organization of F-actin and vimentin was determined by immunostaining. The results of F-actin expression revealed an increase in PDLSCs treated with 100 ng/mL IL-17. Regarding Dox, no changes were observed in F-actin expression compared to the control. However, on the other hand, Dox highly reversed the stimulatory effect of IL-17 on F-actin expression; thus, these results are in line with the effects of IL-17 and Dox on PDLSC migration. On the contrary, when analyzing the expression of intermediate cytoskeletal filament vimentin, immunostaining results revealed that IL-17 slightly decreased the vimentin expression of PDLSCs. The lower vimentin expression pattern was even more pronounced when PDLSCs were treated with Dox alone or combined with 100 ng/mL IL-17.

### 3.3. Dox Inhibits MMP2 Expression of PDLSCs Abrogating the Effect of IL-17

Since MMP2 is shown to be implicated in periodontal tissue degradation [16,22], we aimed to determine whether Dox interferes with IL-17-modulated expression of this proteolytic enzyme. For this purpose, expression of MMP2 was determined by immunofluorescence, evidencing constitutive expression of MMP2 in PDLSCs. Cells treated with IL-17 showed a dose-dependent increase in MMP2 expression, while Dox completely abrogated this effect of IL-17, matching the results concerning PDLSC migration and indicating the MMP2 involvement in cell migration as previously described [23].

**Figure 3:**
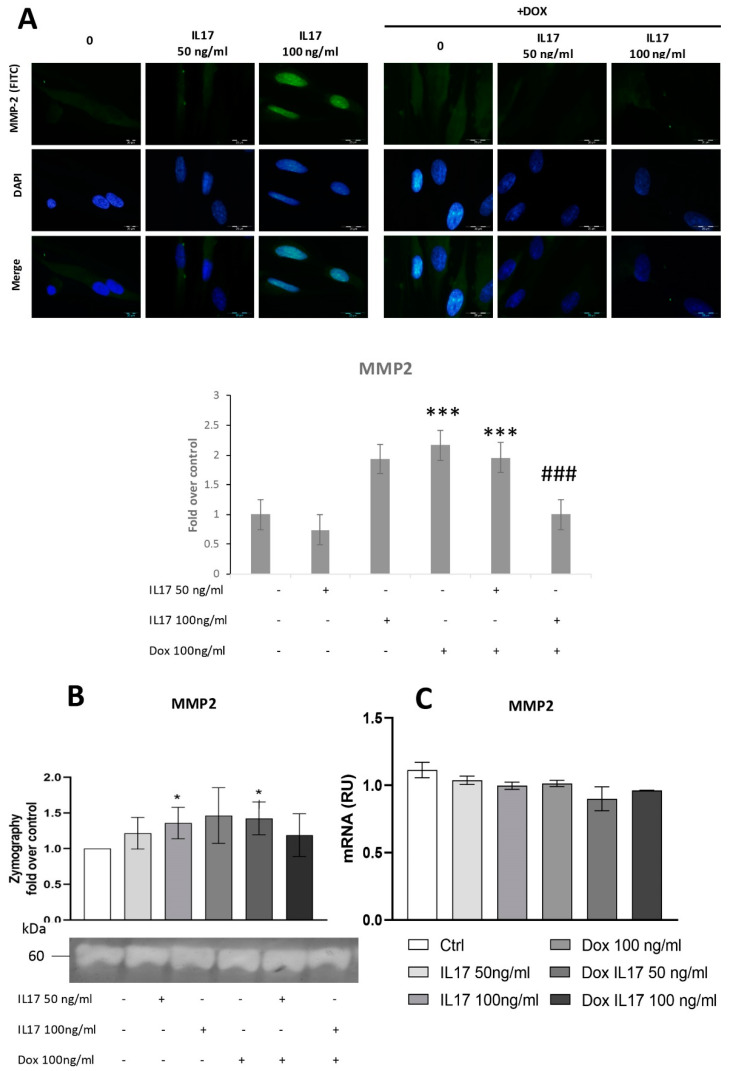
Dox inhibits MMP2 expression of PDLSCs, abrogating the effect of IL-17. The cells were treated for 24 h with 0, 50 and 100 ng/mL IL-17 with or without Dox 100 ng/mL: (**A**) Expression of MMP2 in PDLSCs analyzed by immunostaining with MMP2 primary antibody, corresponding FITC-conjugated secondary antibody and DAPI. Representative images of immunofluorescence microscopy are shown. Scale bars: 20 μm. Graphical: of MMP2 protein expression as analyzed by Image J, relative protein expression normalized to control. Results in graphs are presented as mean ± SEM from at least three independent experiments. Statistically significant differences: *** *p* < 0.001 compared to control, ### *p* < 0.001 compared to Dox. (**B**) MMP2 activity and protein expression were determined by zymography. Statistically significant differences: * *p* < 0.05 compared to control (**C**) MMP2 normalized to the Ct value of the housekeeping gene GAPDH. Results were presented as the mean ± SEM from at least two independent experiments.

To test the activity of MMP2 secreted into conditioned media, a zymography test was performed, as previously described [20]. The obtained results demonstrated that IL-17 induced a mild but statistically significant dose-dependent increase in MMP2 production and activity in PDLSCs (Figure 3B). On the other hand, while Dox alone stimulated MMP2 production by PDLSCs, it completely reversed the effect of IL-17 as decreased MMP2 expression was evidenced in PDLSCs treated with both IL-17 and Dox. However, when MMP2 expression was analyzed at the gene level by qRT-PCR, no significant changes in MMP2 mRNA level were detected for IL-17- and Dox-treated PDLSCs compared to the nontreated control (Figure 3C).

### 3.4. Dox Stimulates Osteogenic Differentiation of PDLSCs Annulling the Inhibitory Effect of IL-17 on PDLSCs Osteogenesis

**Figure 4:**
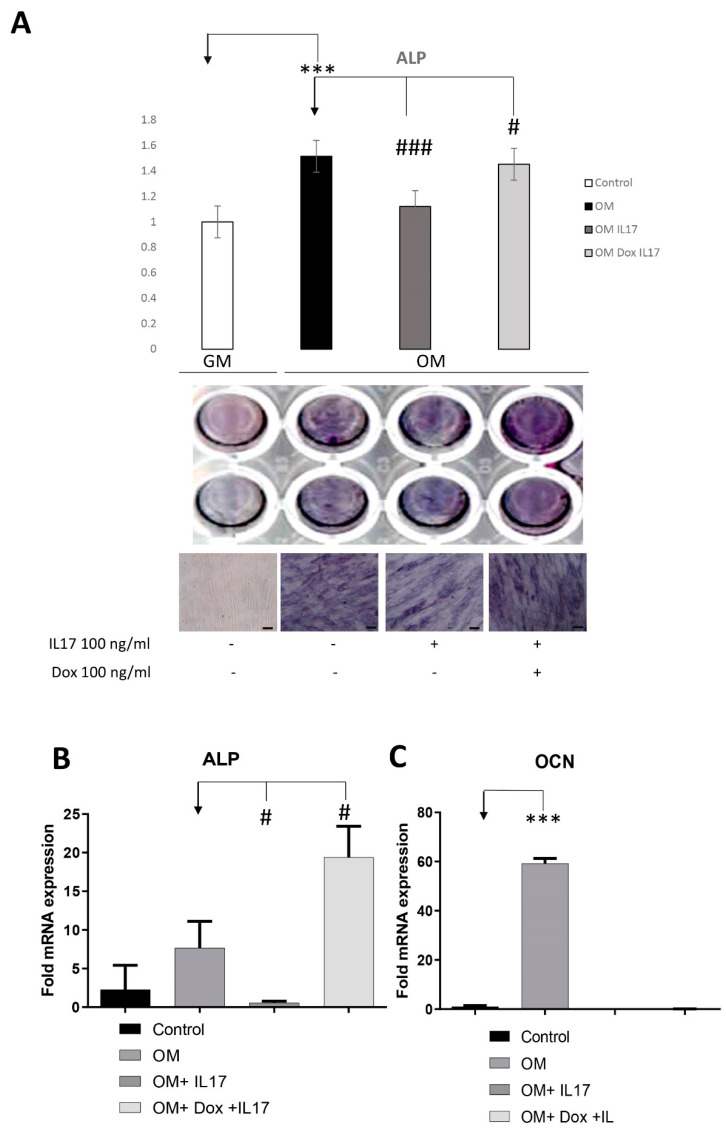
Effects of IL-17 and Dox on PDLSCs osteogenic differentiation. Cells were pretreated with IL-17 (100 ng/mL) for 3 days and afterward cultivated in GM or OM with or without Dox (100 ng/mL). (**A**) ALP activity in tested PDLSCs was examined, and quantified values expressed relative to untreated cells, to which an arbitrary value of 1 was given. Means ± SEM values from at least three independent experiments are presented. Significant difference from the control (indicated by arrows) by *t*-test: *** *p* < 0.001, # *p* < 0.05 ### *p* < 0.001. Scale bars: 50 μm. Graphical presentation of ‘osteogenic differentiation markers’ mRNA expression analyzed by real-time qPCR: relative gene expression levels for (**B**) ALP and (**C**) OCN normalized to the Ct value of the housekeeping gene GAPDH. Results were presented as the mean ± SEM from at least two independent experiments. Statistically significant differences from the control (indicated by arrows): # *p* < 0.05, *** *p* < 0.001 compared to control.

To analyze whether IL-17 affects PDLSCs osteogenic differentiation, cells were pretreated with IL-17 (0 and 100 ng/mL) for three days. Upon pretreatment, PDLSCs were cultivated for the next seven days in GM (control) or OM in the presence or absence of Dox (0 and 100 ng/mL). Afterward, cells were subjected to the determination of an early osteogenic marker, ALP. As shown in Figure 4A, IL-17 significantly decreased early osteogenic differentiation of PDLSCs since reduced levels of ALP activity were shown in IL-17-pretreated cells subjected to osteogenic differentiation (OM). Moreover, Dox annulled the effect of IL-17 pretreatment, causing a statistically significant increase in ALP levels of PDLSCs cultured in OM, indicating the specific functional activity of Dox regarding osteogenic differentiation and regenerative capacity of PDLSCs.

The results of the observed osteo-inductive effect of Dox were further confirmed by gene expression analyses of osteogenic-related markers assessed by qPCR after the pretreatment with IL-17 (0 and 10 ng/mL) for three days and further cultivation in GM (control) or OM for seven days in the presence or absence of Dox (100 ng/mL). Statistically significant inhibition of ALP and Ocn gene expression was detected upon osteogenic induction (OM) in PDLSCs pretreated with IL-17. In addition, Dox completely reversed the effect of IL-17 pretreatment upregulating the gene expression of ALP in PDLSCs cultured in OM after IL-17 treatment. On the other hand, the gene expression level of the late osteogenesis marker, OCN, was reduced in IL-17-pretreated PDLSCs either cultured with or without Dox in OM.

### 3.5. Impact of IL-17 and Dox on PDLSC Mitochondrial Bioenergetics

**Figure 5:**
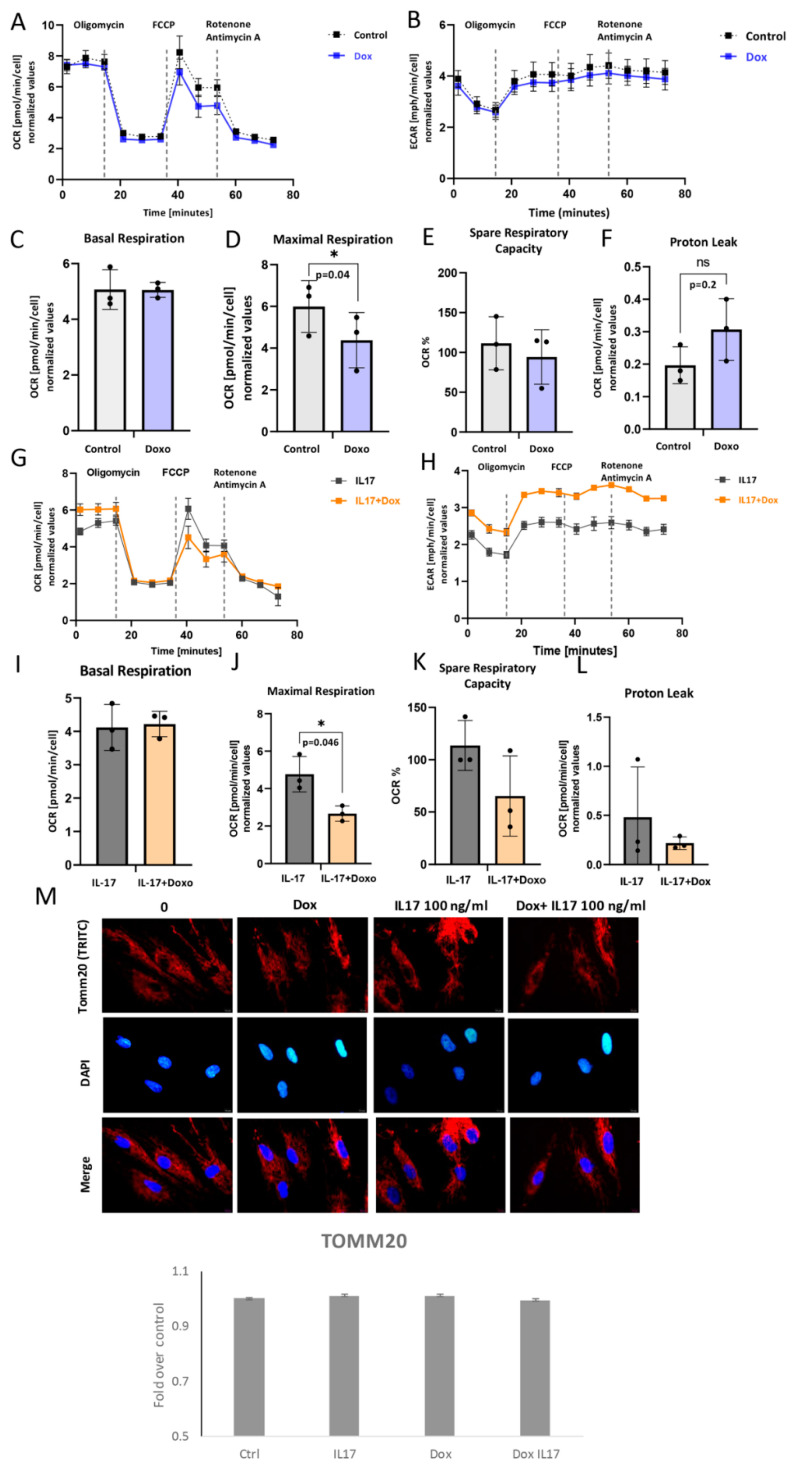
Mitochondrial respiration capacity: (**A**,**G**) Dynamics of OCR and (**B**,**H**) ECAR: Seahorse extracellular flux assay results showing cell responses to the addition of oligomycin, FCCP and Rotenone/Antimycin in presence or absence of IL-17 and/or Doxycycline (Dox). (**C**,**I**) Basal respiration, (**D**,**J**) maximal respiration, (**E**,**K**) spare respiratory capacity and (**F**,**L**) proton leak in PDL cells. Results are presented as mean ± SEM. (**M**) Representative immunofluorescence microscopy images of outer mitochondrial membrane receptor TOMM20. Scale bars 10 μm. Graphical presentation of TOMM20 expression as analyzed by Image J, relative protein expression normalized to control. Results in graphs are presented as mean ± SEM from at least three independent experiments.

In order to understand the underlying metabolic energy profile and mitochondrial function in PDLSCs exposed to Dox and IL-17, the Mito Stress Test was performed. The results showed the attenuated OCR, as well as ECAR to a certain extent, in Dox-exposed PDLSCs in comparison to untreated cells (Figure 5A,B). While basal respiration was similar (Figure 5C), maximal respiration as a maximum capacity of the electron respiratory chain (Figure 5D) and spare respiratory capacity (Figure 5E) were significantly lower in Dox-treated PDLSCs compared to untreated control. However, changes in proton leak were not significant (Figure 5F). Additionally, this was associated with lowered protein expression of TOMM20 (Figure 5M). These findings imply that Dox can impair mitochondrial functionality and biogenesis [24] in PDLSCs.

The impact of Dox on IL-17-exposed PDLSC was also analyzed. The results showed the attenuated OCR (Figure 5G), as well as ECAR (Figure 5H), in PDLSC exposed to Dox+IL-17 when compared to IL-17-treated cells. (Figure 5A). Similarly, together with IL-17, Dox significantly reduced maximal respiration when compared to IL-17-treated PDLSCs (Figure 5J), while no significant effects were found in terms of basal respiration (Figure 5I), spare respiratory capacity (Figure 5K) or proton leak (Figure 5L). Interestingly, Dox diminished IL-17-stimulated mild TOMM20 expression in PDLSCs when compared to untreated control, although not substantially (Figure 5M). Taken together, our results suggest that Dox can significantly reduce mitochondrial-based metabolism in PDLSC in both intact and IL-17-exposed PDLSCs. Furthermore, this implies that Dox is a potent modulator of oxidative phosphorylation and, therefore, important energy-demanding stem cell activities, such as differentiation and migration.

### 3.6. Dox Alters the Expression of Inflammatory Factors of PDLSCs Treated with IL-17

**Figure 6:**
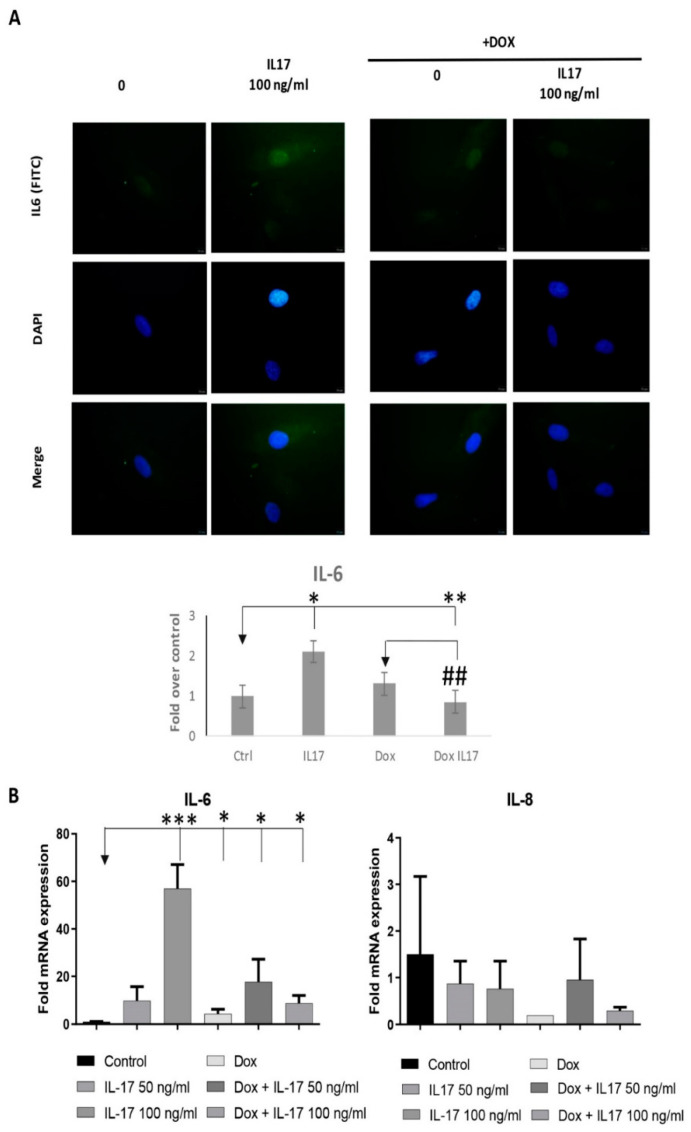
Dox alters the expression of inflammatory factors of PDLSCs treated with IL-17: (**A**) PDLSCs were treated with IL-17 (0, 50 and 100 ng/mL) in presence or absence of Dox (100 ng/mL) for three days. Afterward, cells were immunostained with IL-6 primary antibody, corresponding FITC-conjugated secondary antibody and DAPI. Representative images of cells examined by immunofluorescence microscopy are shown. Scale bars: 10 μm. Graphical presentation of IL-6 protein expression as analyzed by Image J, relative protein expression normalized to control. Results in graphs are presented as mean ± SEM from at least three independent experiments. Statistically significant differences: * *p* < 0.05, ** *p* < 0.01, *** *p* < 0.001 compared to control, ## *p* < 0.01 compared to Dox. (**B**) Graphical presentation of IL-6 and IL-8 mRNA expression as analyzed by qPCR: relative gene expression levels for (**B**) IL-6 and IL-8 normalized to the Ct value of the housekeeping gene GAPDH. Calculations were performed by applying the 2^−ΔΔ*C*T^ method, and results were presented as the mean ± SEM from at least two independent experiments. Statistically significant differences: * *p* < 0.05, ** *p* < 0.01, *** *p* < 0.001 compared to control.

Since numerous discoveries demonstrate mitochondria as pivotal triggers of inflammation being able to intensify the inflammatory response in front of different stimuli [25], our next step was to determine if IL-17 and Dox can alter the expression of proinflammatory factors, such as IL-6 and IL-8 in PDLSCs. Indeed, our results showed that IL-17 increased the expression of IL-6 both at the protein (Figure 6A) and gene level (Figure 6B), as determined by qRT-PCR and immunofluorescence staining, respectively. Additionally, we demonstrated that Dox annulled these effects of IL-17 on IL-6 protein and gene expression in PDLSCs. On the contrary, as for the IL-8 gene expression analyses, our results demonstrated decreased IL8 mRNA expression in both PDLSCs treated with Dox and Dox+IL-17.

## 4. Discussion

The biological potential of Dox in regenerative medicine has not been sufficiently investigated. Although the positive effect of Dox on wound healing has already been shown [5], the effects of Dox on the functional properties of PDLSCs have not been previously tested in the context of an inflammatory environment, such as PD-affected tissue. Therefore, this study aimed to understand the wound-healing and pro-regenerative potential of Dox by analyzing its effects on the functional properties of PDLSCs modulated by IL-17 as an inflammatory mediator of PD. For that purpose, we studied whether Dox can modulate IL-17-affected PDLSCs proliferation, migration and MMP2 expression in parallel with osteogenic differentiation, metabolic activity and proinflammatory immunomodulatory secretory factors expression, including IL-6 and IL-8.

Wound healing is achieved through three different but overlapping phases: proliferation, inflammation and remodeling of affected tissue [5]. Data related to the effects of Dox on the proliferation of MSCs are scarce, and their variety depends on the tissue source of the cells as well as the dosage [26]. Until now, it has been determined that at a low dosage regimen (1 μg/mL), Dox influenced the proliferation and osteogenic capability of bone marrow MSCs derived from streptozotocin-induced diabetic rats [27]. Our results revealed equivalent metabolic activity between treatments with IL-17 and Dox, while both IL-17 and Dox stimulated PDLSC migration when used alone. Interestingly, Dox and IL-17 decreased the motility of PDLSCs in a dose-dependent manner. We cannot exclude the possibility that the inhibitory effect of IL-17 + Dox on PDLSCs migration could be a consequence of the reduced proliferation rate detected by slightly lower proliferation marker Ki67 expression in PDLSCs. However, additional experiments are needed to elucidate this mechanism of cell cycle regulation. To further clarify how IL-17 and Dox interact, modulating PDLSC migration, we next analyzed the expression and organization of cytoskeletal components as important denominators of cell motility. F-actin is known as a cell movement initiator and active regulator of cell polarization [28]. The findings of higher expression of F-actin detected in PDLSCs treated with IL-17 that was abrogated by Dox treatment were in agreement with the results obtained for cell migration of PDLSCs and can also be in correlation with the stress-related state of PDLSCs. Further, Dox additionally reduced protein expression of intermediate filament vimentin in PDLSCs treated with IL-17, which is in line with the results concerning cell migration and F-actin expression. Additionally, recent studies revealed novel functions of vimentin related to migration that should be elucidated in future studies, such as the determination of cellular polarity, regulation of cell contact formation, and arrangement and transport of signal proteins involved in cell motility [28]. However, additional investigations are necessary to define the effects of Dox on cytoskeletal proteins involved in tissue remodeling, such as vitronectin and tubulin. Nevertheless, since Dox induced the inhibition of cell migration, as well as F-actin and vimentin expression in PDLSCs treated with IL-17, it can be speculated that Dox might be a modulator of motility in inflammatory conditions.

Numerous studies describe MMPs as key regulators of ECM degradation in healthy and disease-affected environments [29]. Increased activity of the ECM remodeling proteins MMP2/MMP9 is often associated with periodontal disease [22], where this activity is coordinately regulated by gene expression and controlled enzymatic activity by a wide range of cytokines and growth factors [30]. Our previous studies revealed that IL-17 potentially contributes to ECM degradation in periodontal ligament by stimulating MMP2 gene expression and activity in PDLSCs [16]. This is in line with our previous results; after confirming that IL-17 exerts a stimulatory effect on MMP2 expression in PDLSCs, we determined that Dox abrogated the IL17-stimulated MMP2 expression at the protein level. However, no significant changes were found for MMP2 expression at the gene level in PDLSCs for each treatment tested, although Dox slightly reduced MMP2 mRNA expression in IL-17 treated cells. Numerous literature data determined posttranscriptional and posttranslational regulatory networks influencing MMP protein expression/activity [31,32]. Thereby, it is possible that IL-17- and Dox-modulated MMP2 expression includes posttranslational regulatory mechanisms. Nevertheless, these results indicated that Dox might restrict PDLSC migration in inflammatory conditions through the regulation of cytoskeletal proteins and MMP2.

Next, we aimed to investigate whether Dox can alter other aspects of PDLSCs’ stem cell capacity, including their osteogenic differentiation potential. Tetracyclines were shown to prevent bone loss [33,34]. However, since their effect on osteogenic differentiation of osteoblast lineage cells is controversial [35,36], it has been suggested that the prevention of bone loss by tetracyclines is mainly due to the suppression of osteoclastic bone resorption and not the enhancement of osteoblastic bone formation [37]. Nevertheless, in terms of PDLSCs, it has already been demonstrated that Dox increases osteoblast numbers and decreases osteoclast numbers in the rat model of bone repair [27], once more pointing out that the alteration of pro-regenerative effects of Dox can depend on the MSCs tissue-specific origin. Our results show that Dox increased ALP activity in PDLSCs cultivated under osteogenic conditions, which is in line with the literature data showing that Dox induces bone repair in rats [38]. Importantly, for the first time here, we provide evidence of the Dox potential to annul the inhibitory effect of IL-17 previously demonstrated in our results [17]. These findings indicated the specific functional activity of Dox with regard to osteogenic differentiation and regenerative capacity of PDLSCs. The results of the observed osteoinductive effect of Dox after 7 days at a level of ALP activity were further confirmed at the gene expression level, showing that Dox induced an increased level of ALP gene expression as an essential marker of early osteogenic differentiation, while no effect on the OCN marker of late osteogenic maturation was observed. However, future studies are needed to define the underlying mechanisms of Dox-regulated osteogenic differentiation of PDLSCs in inflammatory conditions by analyzing their effects on later phases of osteogenesis and comparing results with PD-patient-derived cell data. Although Dox limited PDLSC motility, it was observed that the osteogenesis-promoting activity of Dox overcomes the inhibitory effects of IL-17 in PDLSCs, making it a promising candidate in the treatment of inflammation-related bone degradation. Further studies to test the regenerative capacity of PDLSCs should also include quantifying mineralization, cell telomerase activity and the cell cycle since these processes highly regulate cell fate throughout the differentiation process. Also, it would be interesting to compare the effects of Dox on the osteogenic potential of PDLSCs with other osteogenic factors such as Platelets concentrates [39].

Mitochondria play key roles in cellular energy metabolism by generating most of the intracellular adenosine triphosphate (ATP) in the process of mitochondrial respiration. Present comprehension of stem cell bioenergetics favors glycolysis over oxidative phosphorylation as a common mechanism to induce stemness in cells in vivo [40]. During the energy-demanding process of bone formation, proliferative osteoblastic cells use glycolysis in vitro and in vivo [41]. For the first time, our results showed reduced OCR as well as ECAR in PDLSCs exposed to Dox either co-treated with IL-17 or not in comparison to corresponding control (untreated) cells. As it is known that antibiotic compound such as tetracyclines (Dox) impairs mitochondrial biogenesis and oxidative metabolism [42], we assumed that Dox might also affect mitochondrial functionality in PDLSCs. Indeed, Dox diminished IL-17-stimulated TOMM20 expression in PDLSCs in comparison with the untreated control, and together with the Seahorse results, suggests that Dox can significantly reduce mitochondrial-based metabolism in PDLSC in both intact and IL-17-exposed PDLSCs. In addition, it is possible that decreased oxidative metabolism is implicated in the Dox-mediated reduction in PDLSC motility [43]. However, additional studies should reveal specific mechanisms of Dox-mediated mitochondria metabolism.

Both acute and chronic inflammatory conditions have also recently been shown to affect mitochondrial function [44]. Data obtained in our study showed that IL-17 increased the protein and gene expression of IL-6 in PDLSCs, while Dox abrogated this effect. IL-6 has been reported to activate AMP-activated protein kinase (AMPK), which is known to regulate mitochondrial biogenesis and autophagy [45]. The possible mechanism of Dox mediating stem cell potential of PDLSCs in inflammatory conditions that should further be studied could include the influence of IL-6 expression on the biogenesis of mitochondria in PDLSCs since there is no information in this respect [46].

Studies determining the potential use of PDLSCs to treat periodontal diseases in animal models are ongoing, and it has already been shown that cell proliferation rates in periodontal ligaments increase during orthodontic treatment after injury [47]. Nevertheless, it is also determined that the proliferation rates are lower in adults and decrease with age, so these cells are like MSCs of different origins, sensitive to specific stimuli. Therefore, it would be interesting to transplant Dox-treated PDLSCs in immunocompromised mice using ‘cell sheet technology’, in which, upon treatment, intact cell sheets can be harvested from temperature-responsive culture dishes and providing the better vascularization of engineered cell sheet constructs [48].

## 5. Conclusions

This study demonstrates the important activity of Dox in the IL-17-affected inflammatory microenvironment of the periodontium, showing its potential to restrict migration and mitochondria oxidative metabolism but support osteogenic potential in PDLSCs. Opening new possibilities for its potential use in regenerative medicine should be taken with precautions since further studies should be performed to discover the mechanisms of Dox mediating mitochondria biogenesis and potential immunoregulatory properties of PDLSCs in order to contribute to the development of translational and clinically acceptable protocols.

## Figures and Tables

**Table 1:** PCR primer sets used in experiments.

Gene	Sequence (5′–3′)
**GAPDH**	F: GAAGGTGAAGGTCGGAGT
	R: GAAGATGGTGATGGGATTTC
**MMP-2**	F: GCCAAGCGTCTAGCAATACC
	R: TCTGGGGCAGTCCAAAGAAC
**IL6**	F: TACCCCCAGGAGAAGATTCC
	R: TTTTCTGCCAGTGCCTCTTT
**IL8**	F: GTG CAG TTTTGC CAA GGA GT
	R: CTC TGC ACC CAG TTT TCC TT
**Osteocalcin**	F: TCACACTCCTCGCCCTATTGG
	R: GGGCAAGGGGAAGAGGAAAGA
**ALP**	F: CACCCACGTCGATTGCATCT
	R: TAGCCACGTTGGTGTTGAGC

## Data Availability

The data presented in this study are available from the corresponding author upon reasonable request.
